# Dental plaque as a biofilm and a microbial community – implications for health and disease

**DOI:** 10.1186/1472-6831-6-S1-S14

**Published:** 2006-06-15

**Authors:** Philip D Marsh

**Affiliations:** 1 Centre for Emergency Preparedness & Response, Salisbury SP4 0JG, and Division of Oral Biology, Leeds Dental Institute, Leeds LS2 9LU, UK

## Abstract

Dental plaque is a structurally- and functionally-organized biofilm. Plaque forms in an ordered way and has a diverse microbial composition that, in health, remains relatively stable over time (microbial homeostasis). The predominant species from diseased sites are different from those found in healthy sites, although the putative pathogens can often be detected in low numbers at normal sites. In dental caries, there is a shift toward community dominance by acidogenic and acid-tolerating species such as mutans streptococci and lactobacilli, although other species with relevant traits may be involved. Strategies to control caries could include inhibition of biofilm development (e.g. prevention of attachment of cariogenic bacteria, manipulation of cell signaling mechanisms, delivery of effective antimicrobials, etc.), or enhancement of the host defenses. Additionally, these more conventional approaches could be augmented by interference with the factors that enable the cariogenic bacteria to escape from the normal homeostatic mechanisms that restrict their growth in plaque and out compete the organisms associated with health. Evidence suggests that regular conditions of low pH in plaque select for mutans streptococci and lactobacilli. Therefore, the suppression of sugar catabolism and acid production by the use of metabolic inhibitors and non-fermentable artificial sweeteners in snacks, or the stimulation of saliva flow, could assist in the maintenance of homeostasis in plaque. Arguments will be presented that an appreciation of ecological principles will enable a more holistic approach to be taken in caries control.

## Introduction

Dental plaque is the community of microorganisms found on a tooth surface as a biofilm, embedded in a matrix of polymers of host and bacterial origin [1,2]. Of clinical relevance is the fact that biofilms are less susceptible to antimicrobial agents, while microbial communities can display enhanced pathogenicity (pathogenic synergism) [3]. The structure of the plaque biofilm might restrict the penetration of antimicrobial agents, while bacteria growing on a surface grow slowly and display a novel phenotype, one consequence of which is a reduced sensitivity to inhibitors [4]. Plaque is natural and contributes (like the resident microflora of all other sites in the body) to the normal development of the physiology and defenses of the host [5].

### Development of dental plaque biofilms

Dental plaque forms via an ordered sequence of events, resulting in a structurally- and functionally-organized, species-rich microbial community [2]. Distinct stages in plaque formation include: acquired pellicle formation; reversible adhesion involving weak long-range physico-chemical interactions between the cell surface and the pellicle, which can lead to stronger adhesin-receptor mediated attachment; co-adhesion resulting in attachment of secondary colonizers to already attached cells (Cisar – this symposium)[6]; multiplication and biofilm formation (including the synthesis of exopolysaccharides) and, on occasion, detachment. The increase in knowledge of the mechanisms of bacterial attachment and co-adhesion could lead to strategies to control or influence the pattern of biofilm formation (Cisar – this symposium). Analogs could be synthesized to block adhesin-receptor attachment or co-adhesion, and the properties of the colonizing surfaces could be chemically modified to make them less conducive to microbial colonization. However, cells can express multiple types of adhesin [7,8], so that even if a major adhesin is blocked, other mechanisms of attachment may be invoked. Furthermore, although adhesion is necessary for colonization, the final proportions of a species within a mixed culture biofilm such as dental plaque will depend ultimately on the ability of an organism to grow and outcompete neighboring cells.

Once formed, the overall composition of the climax community of plaque is diverse, with many species being detected at individual sites. Molecular ecology approaches, in which 16S rRNA genes are amplified from plaque samples, have identified >600 bacterial and *Archae *taxa, of which approximately 50% are currently unculturable [9]. Once plaque forms, its species composition at a site is characterized by a degree of stability or balance among the component species, in spite of regular minor environmental stresses, e.g., from dietary components, oral hygiene, host defenses, diurnal changes in saliva flow, etc. This stability (termed *microbial homeostasis*) is not due to any biological indifference among the resident organisms, but is due to a balance imposed by numerous microbial interactions, including examples of both synergism and antagonism [10]. These include conventional biochemical interactions such as those necessary to catabolize complex host glycoproteins and to develop food chains, but in addition, more subtle cell-cell signalling can occur. This signalling can lead to coordinated gene expression within the microbial community, and these signalling strategies are currently being viewed as potential targets for novel therapeutics [11,12].

### Perturbations to dental plaque

In any ecosystem, microbial homeostasis can break down on occasion due to a substantial change in a parameter that is critical to maintaining ecological stability at a site, resulting in the outgrowth of previously minor components of the community. A clinical consequence of this breakdown in the mouth can be disease.

Significant parameters regulating homeostasis in the mouth include the integrity of the host defenses (including saliva flow) and the composition of the diet [13]. Subjects who regularly consume dietary components with a high fermentable sugar content have greater proportions of mutans streptococci and lactobacilli in plaque, while impairment of neutrophil function is a risk factor for periodontal diseases. Much less is known about the significance of particular antimicrobial peptides in regulating the resident microflora at sites in the body, but a reduction in some of their activities may increase the risk of caries (Dale – this symposium). Certainly, antimicrobial peptides are being recognized as important components in controlling microbial populations in the mouth, although their role is complex because they are multi-functional and have more than a mere antimicrobial action; for example, by linking the innate and adaptive arms of the immune response [14].

In addition, identification of factors that regulate the natural homeostasis present in plaque during health but, when perturbed, drive the enrichment of putative oral pathogens could open up novel ways to control plaque composition. Manipulation of these ecological influences could help maintain the beneficial microbial composition and normal metabolic activity of plaque biofilms, and augment more conventional approaches to control caries. These concepts will be explored throughout the remainder of this paper.

### Dental plaque and disease

Numerous studies have been undertaken to determine the composition of the plaque microflora from diseased sites in order to try and identify those species directly implicated in causing pathology. Interpretation of the data from such studies is difficult because plaque-mediated diseases occur at sites with a pre-existing diverse resident microflora, and the traits associated with cariogenicity (acid production, acid tolerance, intracellular and extracellular polysaccharide production) are not restricted to a single species. A comparison of the properties of strains representing several streptococcal species have shown considerable overlap in the expression of these cariogenic traits [15] (see below). Microorganisms in biofilms such as plaque are in close physical contact, and this can increase the probability of interactions, some of which can modulate the pathogenic potential of cariogenic bacteria (for example, Kuramitsu and Wang – this symposium). Similarly, the consequence of acid production by cariogenic species can be ameliorated by the development of food chains with *Veillonella *spp., or due to base production by neighboring organisms. Not surprisingly, therefore, there has been only limited success in using the presence of specific species as diagnostic or prognostic indicators of disease. The advent of microarrays, in which the presence of all of the possible groups of micro-organisms in plaque can be determined, may enable particular microbial profiles (or molecular "signatures") to be identified that correlate with caries or periodontal disease (Stahl, this symposium), although markers of biochemical activity might also be needed.

Despite all of these issues, clinical studies have shown that caries is associated with increases in the proportions of acidogenic and aciduric (acid-tolerating) bacteria, especially mutans streptococci (such as *S. mutans *and *S. sobrinus*) and lactobacilli, which are capable of demineralizing enamel [16-19]. These bacteria can rapidly metabolize dietary sugars to acid, creating locally a low pH. These organisms grow and metabolize optimally at low pH. Under such conditions they become more competitive, whereas most species associated with enamel health are sensitive to acidic environmental conditions. However, although mutans streptococci are strongly implicated with caries, the association is not unique; caries can occur in the apparent absence of these species, while mutans streptococci can persist without evidence of detectable demineralization [20,13]. Indeed, in such circumstances, some acidogenic, non-mutans streptococci are implicated with disease [18,21,22]. Detailed studies of the glycolytic activity of a large number of oral streptococci have shown that some strains of non-mutans streptococci(e.g. *S. mitis *biovar 1 and *S. oralis*) can still metabolize sugars to acid at a moderately low environmental pH at rates comparable to those achieved by mutans streptococci[15].

## Source of cariogenic pathogens

**Figure 1:**
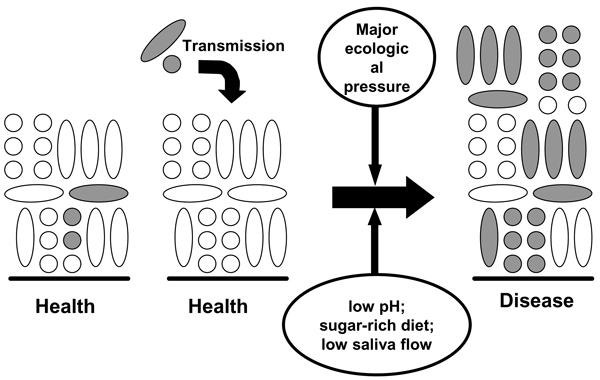
Schematic representation of the relationship between the microbial composition of dental plaque in health and disease. Potential pathogens (grey) may be present in low numbers in plaque, or transmitted in low numbers to plaque; both situations may be compatible with health. A major ecological pressure will be necessary for such pathogens to outcompete other members of the resident microflora (white) and achieve the levels (numerical dominance) needed for disease to occur. Possible ecological pressures for caries include a sugar-rich diet, conditions of low pH, or low saliva flow. Disease could be prevented not only by targeting the pathogen directly (*e.g*. with antimicrobial or anti-adhesion agents) but also indirectly by interfering with the ecological pressure responsible for the selection of the pathogen.

The origin and role of oral pathogens has been the subject of much debate. Indeed, the resolution to this debate is pivotal to the development of effective plaque control strategies. Early studies using conventional culture techniques often failed to recover the putative pathogens from healthy sites or, when pathogens were present, they comprised only a small proportion of the microflora. However, the recent application of more sensitive molecular techniques has led to the frequent detection of low levels of several pathogens (implicated in caries and periodontal diseases) at a wide range of sites [23]. Bacterial typing schemes have shown that identical strains of putative cariogenic bacteria can be found in the plaque of mother (or other close caregiver) and infants [24], implying that transmission of such bacteria can occur. In either situation (*i.e*. natural low levels of "pathogens" or low levels of exogenously-acquired "pathogens"), these species would have to outcompete the already established residents of the microflora in order to achieve an appropriate degree of numerical dominance to cause disease. As argued above, in order for this to happen, the normal homeostatic mechanisms would need to be disrupted, and this is only likely to occur if there is a major disturbance to the local habitat (Figure 1). This suggests that plaque-mediated diseases result from imbalances in the resident microflora resulting from an enrichment within the microbial community of the pathogens due to the imposition of strong selective pressures. If so, interference with these driving forces could prevent pathogen selection and reduce disease incidence.

### Factors responsible for the disruption of microbial homeostasis

Studies of a range of habitats have given clues as to the type of factors capable of disrupting the intrinsic homeostasis that exists within microbial communities. A common feature is a significant change in the nutrient status, such as the introduction of a novel substrate or a major chemical perturbation to the site. For example, in environmental microbiology, it is recognized that nitrogenous fertilizers washed off farmland into lakes and ponds can promote overgrowth by algae. The algae can consume dissolved oxygen in the water, leading to the loss of aerobic microbial, plant, and insect life (eutrophication). Similarly, atmospheric pollution with sulphur dioxide and nitrogen oxides can result in acid rain, causing damage to plants and trees, and loss of aquatic life.

The local environment is known to change in plaque during disease. Caries is associated with a more regular intake of fermentable carbohydrates in the diet, and hence plaque is exposed more frequently to low pH. The effect of such environmental changes in nutrient availability and pH on gene expression by oral bacteria predominating in either health or disease has shown that organisms such as mutans streptococci are better able to adapt to low pH, and they up-regulate a number of genes that protect against acid stress. For example, cells of *S. mutans *up-regulate a number of specific proteins and functions when exposed to sub-lethal pH values (approximately 5.5). This enhances survival under acidic conditions such as those encountered in caries lesions [25-27]. These differences in phenotype will alter the competitiveness of bacteria in plaque. Laboratory modeling studies involving diverse but defined communities of oral bacteria have been performed to answer specific questions concerning the consequence of such changes on the relative competitiveness of individual species and the impact on community stability. Analysis of these studies led to the formulation of an alternative hypothesis relating the role of oral bacteria to dental disease, and the identification of factors that disrupt the natural balance of the resident plaque microflora.

### Impact of environmental change – mixed culture modeling studies

As stated earlier, individuals who frequently consume sugar in their diet generally have elevated levels of cariogenic bacteria such as mutans streptococci and lactobacilli in their plaque, and are at greater risk of dental caries. In animal studies or epidemiological surveys of humans, it can never be determined whether the rise in cariogenic bacteria is due to the sudden availability of sugar *per se *(*e.g*. because of more efficient sugar transport systems in these bacteria), or is a response to the inevitable conditions of low pH following sugar consumption. Exploitation of the unique benefits of parameter control in the chemostat, coupled with the reproducibility of a defined mixed culture inoculum, enabled these linked effects to be separated for the first time [28]. Two mixed culture chemostats were inoculated with 9 or 10 species (representative of those in health and disease) in a growth medium at pH 7.0 in which mucin was the main source of carbohydrate; under these conditions, *S. mutans *and *Lactobacillus rhamnosus *were noncompetitive and made up <1% of the total microflora (Table 1). Once the consortia were stably established, both chemostats were pulsed daily for ten consecutive days with a fermentable sugar (glucose). In one chemostat, the pH was maintained automatically throughout the study at neutral pH (as is found in the healthy mouth) in order to determine the effect of the addition of a fermentable sugar on culture stability, while in the other the pH was allowed to fall by bacterial metabolism for six hours after each pulse (as occurs *in vivo*); the pH was then returned to neutrality for 18 hours prior to the next pulse [28]. Daily pulses of glucose for 10 consecutive days at a constant pH 7.0 had little or no impact on the balance of the microbial community, and the combined proportions of *S. mutans *and *L. rhamnosus *stayed at ca. 1% of the total microflora (Table 1). In contrast, however, when the pH was allowed to change after each pulse, there was a progressive selection of the cariogenic (and acid-tolerating) species at the expense of bacteria associated with dental health. After the final glucose pulse, the community was dominated by species implicated in caries (*S. mutans *and *L. rhamnosus *comprised ca. 55% of the microflora) [28]. When this study was repeated, but the pH fall was restricted after each glucose pulse to either pH 5.5, 5.0, or 4.5 in independent experiments [29], a similar enrichment of cariogenic species at the expense of healthy species was observed, but their rise was directly proportional to the extent of the pH fall (Table 1). Collectively, these studies showed conclusively that it was the low pH generated from sugar metabolism rather than sugar availability that led to the breakdown of microbial homeostasis in dental plaque. This finding has important implications for caries control and prevention; the data suggest that the selection of cariogenic bacteria could be prevented if the pH changes following sugar metabolism could be reduced (see later).

### Current hypotheses to explain the role of plaque bacteria in the etiology of dental caries

There have been two main schools of thought on the role of plaque bacteria in the etiology of caries and periodontal diseases. The "Specific Plaque Hypothesis" proposed that, out of the diverse collection of organisms comprising the resident plaque microflora, only a few species are actively involved in disease [30]. This proposal focused on controlling disease by targeting preventive measures and treatment against a limited number of organisms. In contrast, the "Non-Specific Plaque Hypothesis" considered that disease is the outcome of the overall activity of the total plaque microflora [31]. In this way, a heterogeneous mixture of microorganisms could play a role in disease. In some respects, the arguments about the relative merits of these hypotheses may be about semantics, since plaque-mediated diseases are essentially mixed culture (polymicrobial) infections, but in which only a limited (perhaps specific!) number of species are able to predominate.

**Figure 2:**
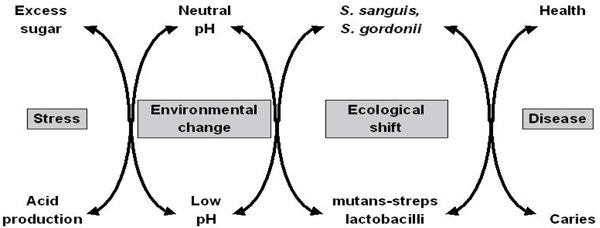
The "ecological plaque hypothesis" and the prevention of dental caries. Caries is a result of changes in the environment due to acid production from the fermentation of dietary carbohydrates, which selects for acidogenic and acid-tolerating species such as mutans streptococci and lactobacilli. Disease could be prevented not only by targeting the putative pathogens directly, but also by interfering with the key environmental factors driving the deleterious ecological shifts in the composition of the plaque biofilms [32].

More recently, an alternative hypothesis has been proposed (the "Ecological Plaque Hypothesis") that reconciles the key elements of the earlier two hypotheses [32]. The data from the mixed cultures studies described above, and from other work, provide an argument for plaque-mediated diseases being viewed as a consequence of imbalances in the resident microflora resulting from an enrichment within the microbial community of these "oral pathogens.". Potentially cariogenic bacteria may be found naturally in dental plaque, but these organisms are only weakly competitive at neutral pH, and are present as a small proportion of the total plaque community. In this situation, with a conventional diet, the levels of such potentially cariogenic bacteria are clinically insignificant, and the processes of de- and re-mineralization are in equilibrium. If the frequency of fermentable carbohydrate intake increases, then plaque spends more time below the critical pH for enamel demineralization (approximately pH 5.5). The effect of this on the microbial ecology of plaque is two-fold. Conditions of low pH favor the proliferation of acid-tolerating (and acidogenic) bacteria (especially mutans streptococci and lactobacilli), while tipping the balance towards demineralization (Figure 2). Greater numbers of bacteria such as mutans streptococci and lactobacilli in plaque would result in more acid being produced at even faster rates, thereby enhancing demineralization still further. Other bacteria could also make acid under similar conditions, but at a slower rate [15]. These bacteria could be responsible for some of the initial stages of demineralization or could cause lesions in the absence of other (more overt) cariogenic species in a more susceptible host. If aciduric species were not present initially, then the repeated conditions of low pH coupled with the inhibition of competing organisms might increase the likelihood of successful colonization by mutans streptococci or lactobacilli. This sequence of events would account for the lack of total specificity in the microbial etiology of caries and explain the pattern of bacterial succession observed in many clinical studies.

Key features of this hypothesis are that (a) the selection of "pathogenic" bacteria is directly coupled to changes in the environment (Figure 2), and (b) diseases need not have a specific etiology; any species with relevant traits can contribute to the disease process. Thus, mutans streptococci are among the best adapted organisms to the cariogenic environment (high sugar/low pH), but such traits are not unique to these bacteria. Strains of other species, such as members of the *S. mitis*-group, also share some of these properties and therefore will contribute to enamel demineralization [15,21,22]. A key element of the ecological plaque hypothesis is that disease can be prevented not only by targeting the putative pathogens directly, *e.g*. by antimicrobial or anti-adhesive strategies, but also by interfering with the selection pressures responsible for their enrichment [32].

In dental caries, regular conditions of sugar/low pH or reduction in saliva flow appear to be primary mechanisms that disrupt microbial homeostasis. Strategies that are consistent with the prevention of disease via the principles of the ecological plaque hypothesis include the following:

(a) Inhibition of plaque acid production, *e.g*. by fluoride-containing products or other metabolic inhibitors. Fluoride not only improves enamel chemistry but also inhibits several key enzymes, especially those involved in glycolysis and in maintaining intracellular pH [33]. Fluoride can reduce the pH fall following sugar metabolism in plaque biofilms, and in so doing, prevent the establishment of conditions that favor growth of acid-tolerating cariogenic species [34].

(b) avoidance between main meals of foods and drinks containing fermentable sugars and/or the consumption of foods/drinks that contain non-fermentable sugar substitutes such as aspartame or polyols, thereby reducing repeated conditions of low pH in plaque.

(c) the stimulation of saliva flow after main meals, *e.g*. by sugar-free gum. Saliva will introduce components of the host response, increase buffering capacity, remove fermentable substrates, promote re-mineralization, and more quickly return the pH of plaque to resting levels.

## Conclusion

The key to a more complete understanding of the role of microorganisms in dental diseases such as caries may depend on a paradigm shift away from concepts that have evolved from studies of classical medical infections with a simple and specific (*e.g*. single species) etiology to an appreciation of ecological principles. The development of plaque-mediated disease at a site may be viewed as a breakdown of the homeostatic mechanisms that normally maintain a beneficial relationship between the resident oral microflora and the host. When assessing treatment options, an appreciation of the ecology of the oral cavity will enable the enlightened clinician to take a more holistic approach and consider the nutrition, physiology, host defenses, and general well-being of the patient, as these will affect the balance and activity of the resident oral microflora. Future episodes of disease will occur unless the cause of any breakdown in homeostasis is recognized and remedied. For example, a side effect of many medications is a reduction in saliva flow. This will deleteriously impact on sugar clearance and buffering ability, thereby favoring the growth of acid-tolerating and potentially cariogenic bacteria. Identification of such critical control points can lead to the selection of appropriate caries preventive strategies that are tailored to the needs of individual patients. In this way, the clinician does not just treat the end result of the caries process, but also attempts to identify and interfere with the factors that, if left unaltered, will inevitably lead to more disease.

## Competing interests

The author(s) declare that they have no competing interests.

## Figures and Tables

**Table 1:** The effect of glucose and low pH on the stability of a microbial community. A mixed culture of 9 oral bacteria (representative of those found in health and disease) were grown in a mucin-based medium at a constant pH 7.0. In independent experiments, the culture was pulsed daily on 10 consecutive days with 28 mM glucose. In one culture the pH was maintained throughout the pulsing at pH 7.0, while in other experiments the pH was allowed to fall by bacterial metabolism for 6 hours to pre-set levels of pH 5.5, 5.0 or 4.5. In a final study, the pH was allowed to fall without any pH control imposed [28,29]. Viable counts were determined on selective and non-selective media; proportions are shown after the final glucose pulse for two species implicated in caries and for two species associated with sound enamel

Bacterium	Percentage of total viable count (%)					
	
	Pre-pulse	pH 7.0	5.5	5.0	4.5	no pH control
	
*Streptococcus mutans*	0.3	1.0	4.2	7.9	9.6	18.9
*Lactobacillus rhamnosus*	0.1	0.2	2.4	6.2	13.9	36.1
*Streptococcus gordonii*	28.3	25.0	2.6	6.9	2.7	0.2
*Fusobacterium nucleatum*	15.2	9.5	7.4	2.3	2.7	<0.001

## References

[B1] Socransky SS, Haffajee AD (2002). Dental biofilms: difficult therapeutic targets. Periodontology.

[B2] Marsh PD (2004). Dental plaque as a microbial biofilm. Caries Res.

[B3] van Steenbergen TJM, van Winkelhoff AJ, de Graaff J (1984). Pathogenic synergy: mixed infections in the oral cavity. Antonie van Leeuwenhoek.

[B4] Gilbert P, Maira-Litran T, McBain AJ, Rickard AH, Whyte FW (2002). The physiology and collective recalcitrance of microbial biofilm communities. Adv Microb Physiol.

[B5] Marsh PD (2000). Role of the oral microflora in health. Microb Ecol Health Dis.

[B6] Kolenbrander PE, Andersen RN, Kazmerak KM, Palmer RJ, Allison DG, Gilbert P, Lappin-Scott HM, Wilson M (2000). Coaggregation and coadhesion in oral biofilms. Community structure and co-operation in biofilms.

[B7] Hasty DL, Ofek I, Courtney HS, Doyle RJ (1992). Multiple adhesins of *streptococci*. Infect Immun.

[B8] Zhang Y, Lei Y, Nobbs A, Khammanivong A, Herzberg MC (2005). Inactivation of *Streptococcus gordonii *SspAB alters expression of multiple adhesin genes. Infect Immun.

[B9] Wade W, Newman HN, Wilson M (1999). Unculturable bacteria in oral biofilms. Dental plaque revisited Oral biofilms in health and disease.

[B10] Marsh PD, Featherstone A, McKee AS, Hallsworth AS, Robinson C, Weatherell JA, Newman HN, Pitter AF (1989). A microbiological study of early caries of approximal surfaces in schoolchildren. J Dent Res.

[B11] Kolenbrander PE, Andersen RN, Blehert DS, Egland PG, Foster JS, Palmer RJ (2002). Communication among oral bacteria. Microbiol Molec Biol Rev.

[B12] Suntharalingam P, Cvitkovitch DG (2005). Quorum sensing in streptococcal biofilm formation. Trends Microbiol.

[B13] Marsh PD (1989). Host defenses and microbial homeostasis: role of microbial interactions. J Dent Res.

[B14] Devine DA (2003). Antimicrobial peptides in defence of the oral and respiratory tracts. Mol Immunol.

[B15] de Soet JJ, Nyvad B, Kilian M (2000). Strain-related acid production by oral *streptococci*. Caries Res.

[B16] Loesche WJ (1986). Role of *Streptococcus mutans *in human dental decay. Microbiol Rev.

[B17] Bowden GH (1990). Microbiology of root surface caries in humans. J Dent Res.

[B18] Marsh PD (1999). Microbiologic aspects of dental plaque and dental caries. Dent Clin North Amer.

[B19] Becker MR, Paster BJ, Leys EJ, Moeschberger ML, Kenyon SG, Galvin JL, Boches SK, Dewhirst FE, Griffen AL (2002). Molecular analysis of bacterial species associated with childhood caries. J Clin Microbiol.

[B20] Bowden GH, Hardie JM, McKee AS, Marsh PD, Fillery ED, Slack GL, Stiles HM, Loesche WJ, O'Brien TC (1976). The microflora associated with developing carious lesions of the distal surfaces of the upper first premolars in 13–14 year old children. Proceedings Microbial Aspects of Dental Caries.

[B21] Sansone C, van Houte J, Joshipura K, Kent R, Margolis HC (1993). The association of mutans streptococci and non-mutans streptococci capable of acidogenesis at a low pH with dental caries on enamel and root surfaces. J Dent Res.

[B22] Brailsford SR, Shah B, Simins D, Gilbert S, Clark D, Ines I, Adama SE, Allison C, Beighton D (2001). The predominant aciduric microflora of root-caries lesions. J Dent Res.

[B23] Tanner AC, Milgrom PM, Kent R, Mokeem SA, Page RC, Riedy CA, Weinstein P, Bruss J (2002). The microbiota of young children from tooth and tongue samples. J Dent Res.

[B24] Tanner AC, Milgrom PM, Kent R, Mokeem SA, Page RC, Liao SI, Riedy CA, Bruss JB (2002). Similarity of the oral microbiota of pre-school children with that of their caregivers in a population-based study. Oral Microbiol Immunol.

[B25] Welin J, Wilkins JC, Beighton D, Wrzesinski K, Fey SJ, Mose-Larsen P, Hamilton IR, Svensater G (2003). Effect of acid shock on protein expression by biofilm cells of *Streptococcus mutans*. FEMS Microbiol Lett.

[B26] Len AC, Harty DW, Jacques NA (2004). Stress-responsive proteins are upregulated in *Streptococcus mutans *during acid tolerance. Microbiology.

[B27] McNeill K, Hamilton IR (2004). Effect of acid stress on the physiology of biofilm cells of *Streptococcus mutans*. Microbiology.

[B28] Bradshaw DJ, McKee AS, Marsh PD (1989). Effects of carbohydrate pulses and pH on population shifts within oral microbial communities *in vitro*. J Dent Res.

[B29] Bradshaw DJ, Marsh PD (1998). Analysis of pH-driven disruption of oral microbial communities *in vitro*. Caries Res.

[B30] Loesche WJ (1976). Chemotherapy of dental plaque infections. Oral Sci Rev.

[B31] Theilade E (1986). The non-specific theory in microbial etiology of inflammatory periodontal diseases. J Clin Periodontol.

[B32] Marsh PD (2003). Are dental diseases examples of ecological catastrophes?. Microbiology.

[B33] Marquis RE, Clock SA, Mota-Meira M (2003). Fluoride and organic weak acids as modulators of microbial physiology. FEMS Microbiol Rev.

[B34] Bradshaw DJ, Marsh PD, Hodgson RJ, Visser JM (2002). Effects ofglucose and fluoride on competition and metabolism within *in vitro *dental bacterial communities and biofilms. Caries Res.

